# Host Restrictions of Avian Influenza Viruses: *In Silico* Analysis of H13 and H16 Specific Signatures in the Internal Proteins

**DOI:** 10.1371/journal.pone.0063270

**Published:** 2013-04-30

**Authors:** Ragnhild Tønnessen, Anna G. Hauge, Elisabeth F. Hansen, Espen Rimstad, Christine M. Jonassen

**Affiliations:** 1 Department of Food Safety and Infection Biology, Norwegian School of Veterinary Science, Oslo, Norway; 2 Department of Laboratory Services, Norwegian Veterinary Institute, Oslo, Norway; Thomas Jefferson University, United States of America

## Abstract

Gulls are the primary hosts of H13 and H16 avian influenza viruses (AIVs). The molecular basis for this host restriction is only partially understood. In this study, amino acid sequences from Eurasian gull H13 and H16 AIVs and Eurasian AIVs (non H13 and H16) were compared to determine if specific signatures are present only in the internal proteins of H13 and H16 AIVs, using a bioinformatics approach. Amino acids identified in an initial analysis performed on 15 selected sequences were checked against a comprehensive set of AIV sequences retrieved from Genbank to verify them as H13 and H16 specific signatures. Analysis of protein similarities and prediction of subcellular localization signals were performed to search for possible functions associated with the confirmed signatures. H13 and H16 AIV specific signatures were found in all the internal proteins examined, but most were found in the non-structural protein 1 (NS1) and in the nucleoprotein. A putative functional signature was predicted to be present in the nuclear export protein. Moreover, it was predicted that the NS1 of H13 and H16 AIVs lack one of the nuclear localization signals present in NS1 of other AIV subtypes. These findings suggest that the signatures found in the internal proteins of H13 and H16 viruses are possibly related to host restriction.

## Introduction

Influenza A viruses (family *Orthomyxoviridae*) have a genome of eight negative sense single-stranded RNA segments that encodes up to 13 proteins [1]. To date, 16 hemagglutinin (HA) (H1-H16) and 9 neuraminidase (NA) (N1-N9) subtypes have been detected in wild birds [2], [3]. Ducks, gulls and shorebirds constitute the primary natural reservoir of avian influenza viruses (AIVs) [2], [3]. Influenza A viruses that cause disease in humans and animals have evolved from AIVs [2].

Limited overlap between the flyways used by migratory birds in Eurasia and America has led to the development of two major phylogenetic AIV lineages [4]. AIVs of the H13 and H16 subtypes are mainly detected in gulls, and for these viruses there are separate American and Eurasian phylogenetic lineages [4], [5]. Genetic reassortment frequently occurs for AIVs within continents. More rarely, birds move single or multiple viral segments across continents [5]–[13]. Nevertheless, the number of intercontinental reassortment events found among H13 and H16 AIVs is relatively high [5], [14], and long-distance migrating gull species are believed to play an important role as vectors for geographically reassorted viruses [5]. Eurasian gene segments are often detected in influenza viruses from gulls in America [5], [12]. On the contrary, American gene segments have only occasionally been found in AIVs from gulls in Eurasia [7], [14]. However, the total number of sequenced AIV genomes from gulls in Eurasia is still low [5].

Gulls may get infected by most AIV subtypes found in waterfowl [15]. In contrast, H13 and H16 viruses are extremely rarely detected in surveillance samples from waterfowl [16], and experimentally challenged ducks are resistant to several strains of H13 AIVs [17], [18]. The molecular basis for this host restriction is only partially understood.

HA is an important determinant of host specificity. The HA glycoprotein must interact with a cellular receptor for the virus to get endocytosed. AIVs preferentially bind to Neu5Ac α2-3Gal-terminated receptors [19]. Influenza viruses from ducks and gulls differ in their receptor specificity [20], also in their ability to recognize the more distal parts of the receptor [19]. Several amino acid residues in the receptor-binding site of HA are shared by H13 and H16 AIVs, but not by influenza viruses of other subtypes [3], [20].

Host range is a polygenic trait [2] and previous studies have suggested that several amino acids also in the internal viral proteins of H13 AIVs are associated with host adaptation [2], [21]–[25]. The majority of these studies were performed in the 1990s and the early 2000s on a limited number of sequences from a restricted geographical area. But in a recent study, the occurrence of specific signatures in the internal viral proteins of both H13 and H16 viruses was examined [26]. However, the analysis was performed on American H13 and H16 strains, and only one H16 virus was included. The amount of available AIV sequences has increased vastly the last decade [5]. Therefore, to gain more insight about host restriction of both H13 and H16 AIVs, and to provide a basis for functional studies, we reexamined these AIV subtypes for H13 and H16 specific signatures in the internal viral proteins, using a bioinformatics approach.

In the present study, whole genome sequence data from Eurasian gull AIVs (subtypes H13 and H16) were compared with a selection of Eurasian non H13 and H16 AIVs to search for H13 and H16 specific amino acids in the internal proteins. Five AIV genomes from Norway and 10 AIV genomes from the Influenza Virus Resource were included in this initial analysis. To verify the identified amino acids as H13 and H16 specific signatures, they were checked against a comprehensive set of AIV sequences in GenBank, including all available H13 and H16 sequences [27], [28]. To search for potential functions associated with the identified signatures, *in silico* analyses, including protein similarity searches and prediction of subcellular localization signals were performed. The obtained results suggest that signatures found in the internal proteins of H13 and H16 viruses may be related to host restriction.

## Materials and Methods

### Ethics statement

The samples characterized in this study were obtained from the Norwegian surveillance program for AIV in wild birds. All samples were collected by voluntary, licensed hunters from hunter-harvested birds during the permitted hunting season. No special permit is required during this period.

### Virus isolates

Virus isolates were obtained from samples collected through the surveillance program for AIV in wild birds in Norway during the period 2006–2009. Cloacal and tracheal swabs were collected by hunters from hunter-harvested birds during the ordinary hunting season (August – December). The samples were sent to the Norwegian Veterinary Institute where they were analyzed as previously described [15], [29]. In brief, RNA was extracted [15] and analyzed for AIV by real-time RT-PCR. AIV positive samples were HA and NA subtyped by sequencing of HA2 [30] and full-length NA genes [31]. Virus isolation was performed in 10-day-old embryonated chicken eggs for a selection of real-time RT-PCR positive samples [32], [33]. Virus isolates were stored at −80°C.

Five virus isolates, all originating from samples collected in Rogaland county on the south-west coast of Norway, were used in the study. One H13, one H16 and one non H13/H16 isolate from common gull (*Larus canus*) were chosen: A/common gull/Norway/1313/2009(H13N2), A/common gull/Norway/1617/2006(H16N3) and A/common gull/Norway/1602/2009(H6N8). In addition, two randomly selected AIVs from mallard were included: A/mallard/Norway/779/2009(H3N8) and A/mallard/Norway/1537/2009(H9N2). Allantoic fluids from first (H6, H13), second (H3, H16) and third (H9) egg passages were used for sequencing.

### Sequencing and sequence analysis

For full length amplification of the viral segments, a two-step RT-PCR was performed, using SuperScript® III (Invitrogen, Carlsbad, CA, USA) and the Advantage 2 PCR kit (Clontech, Mountain View, CA, USA). Cycling conditions [29] and primers [31] used were as previously described. For some polymerase basic protein 2 (PB2) and polymerase basic protein 1 (PB1) genes, additional primers (Table S1) were used to obtain full length sequences. Amplicons were purified, using Zymoclean™ Gel DNA recovery kit (Zymo Research, Orange, CA), and cloned into pCR2.1 TOPO vectors (Invitrogen). Sequencing was performed by a commercial sequencing service (GATC Biotech, Constance, Germany). For large amplicons, primers were designed for internal sequencing (Table S2). For each viral segment, three to six clones were sequenced. Initial sequence analysis, including translation of nucleotide sequences into putative amino acid sequences, was performed using Vector NTI Advance ver.11 (Invitrogen). All sequences have been submitted to the EMBL Nucleotide Sequence Database under the accession numbers: HE802704-HE802743.

### Available complete AIV genomes from gulls in Eurasia

A search in the Influenza Virus Resource database [28] for complete AIV genomes from gulls in Europe and Asia identified 13 genomes (as of February 2012) (Table S3). Three of these were excluded from further analysis due to subtype (HPAI H5N1), suboptimal sequence quality (A/slaty-backed gull/Japan/6KS0191/2006(H4N8)) or because it was genetically distinct from all the other Eurasian avian sequences (A/slaty-backed gull/Japan/6KS0185/2006(H4N8)) [34].

### Initial characterization of potentially H13 and H16 specific amino acids

To generate hypotheses regarding the existence of H13 and H16 specific amino acids in the internal viral proteins of H13 and H16 AIVs, the deduced amino acid sequences from the 15 Eurasian viral genomes (Table S3) were aligned using Clustal W in MEGA 4 (data not shown) [35]. The full-length sequences from the following internal viral proteins were analyzed: PB2, PB1, polymerase basic 1 frame 2 (PB1-F2), polymerase acidic (PA), nucleoprotein (NP), matrix 1 (M1), matrix 2 (M2), non-structural protein 1 (NS1) and nuclear export protein (NEP). In the initial analysis, the sequences from each protein were sorted into two groups called Eurasian avian and Eurasian gull AIVs. Eurasian avian viruses were defined as Eurasian AIVs of all subtypes except H13 and H16, while Eurasian gull viruses were defined as Eurasian AIVs from gulls of the H13 and H16 subtypes. All amino acids displaying heterogeneity between these two groups were plotted in Excel spread sheets (Microsoft Excel® 2010, Microsoft Corporation). Amino acids showing a distinct pattern between the Eurasian avian and the Eurasian gull viruses were identified as potentially H13 and H16 specific amino acids (Figures S1–S9) and further analyzed.

### Signature validation

To confirm or reject the initially identified amino acids (Figures S1–S9) as H13 and H16 specific signatures, similarity searches were performed on the protein sequences from the two Norwegian H13 and H16 isolates, using tblastn with up to 10000 target sequences of influenza A virus from any species [36]. The target sequences were converted to FASTA format using FASTA BLAST Scan 2.1 [37], and the potentially H13 and H16 specific amino acids (Figures S1–S9) were compared to the amino acids of the target sequences using BioEdit version 7.1.3 [38]. Amino acids mostly or entirely found in H13 and H16 AIVs, were defined as H13 and H16 specific signatures. If several consecutive or nearby amino acids seemed to be involved in a longer motif they were searched together, including any amino acids positions in between, to identify larger H13 and H16 specific motifs where single amino acid positions would be missed when searched individually. The analysis was repeated on all AIV sequences available (after collapsing identical sequences) at the NCBI Influenza Virus Resource as of April 2012. AIV sequences of all subtypes and from all avian species, both wild and domestic, were included in this search. Furthermore, all H13 and H16 AIV sequences available at the Influenza Virus Resource as of August 2012 were used to finally verify the signatures, and to determine if different signatures were present in H13 and H16 AIVs from Eurasia and America. AIV proteins encoded by reassorted gene segments that most likely originated from subtypes other than H13 and H16, [5], were ignored for the purpose of the latter analysis.

### Prediction of protein similarity and localization

In order to search for potential functions of the verified H13 and H16 specific signatures, protein motif and similarity searches were performed for each of the internal gene products of A/common gull/Norway/1313/2009(H13N2), A/common gull/Norway/1617/2006(H16N3) and A/mallard/Norway/779/2009(H3N8), using Motif search [39], including PROSITE, ProDom, BLOCKS, PRINTS and Pfam. The sequences were compared to previously characterized proteins. When protein family signatures were identified in H13 and H16 viruses but not in AIVs of other subtypes, using A/mallard/Norway/779/2009(H3N8) as reference, the searches were repeated with *in silico* modifications of the H13 and H16 specific signatures to confirm H13 and H16 specific amino acid motifs associated with the proposed protein family signatures. Further, prediction of subcellular localization signals within the amino acid sequences was assessed using PSORT [40].

## Results

### Amino acids initially characterized as potentially H13 and H16 specific

Among the 15 AIV genomes initially analyzed for amino acid heterogeneity in the internal proteins, the highest percentage of variable amino acid sites was found in PB1-F2 (54.4%), followed by NS1 (29.6%), NEP (14.0%), M2 (12.4%), NP (6.6%), PB2 (5.9%), PA (4.9%), PB1 (4.2%) and M1 (2.4%). All amino acid positions in the internal proteins that displayed variation between the 15 Eurasian avian and Eurasian gull AIVs included in the initial analysis are shown in Figures S1–S9.

### H13 and H16 specific amino acid signatures

Several of the amino acids assumed to be H13 and H16 specific in the initial analysis were rejected as signatures after validation against the sequences in the NCBI database. Among the verified signatures, some were mostly or entirely found in H13 and H16 AIVs, while others were primarily identified in H13 and H16 AIVs either from Eurasia or America. A few signatures were only present in subgroups of Eurasian or American gull viruses. The amino acids in the internal AIV proteins that were finally verified as H13 and H16 specific signatures are listed in Table 1. H13 and H16 specific signature variants that were only found among the NCBI sequences, and not among the 15 initially analyzed sequences, were also included in Table 1.

**Table 1:** Amino acid signatures found mostly or entirely in the internal proteins of Eurasian and American H13 and H16 AIVs from gulls and shorebirds, validated against a comprehensive set of AIV sequences in GenBank.

Protein	Amino acid position(s)	Signatures^1,2^	
		Eurasian H13, H16	American H13, H16
PB2	470	N	N
PB2	559–560	VI/(FI)	MI
PB2	674	S	S
PB1	391	(N)	
PB1-F2	33–37	(LSLTR/LSLTL)	
PA	387–388	(IN)	
NP	87	R	R
NP	105–109	VRELV	VRELV
NP	269	A	A
NP	350–353	ARVL	TRVL
NP	375	S	N
NP	432–433	(SA)	(NP)
M1	63	(I)	
M1	207	G	(G)
M2	55–58	IKYE/IKYD	
NS1	52	I/M	I/M
NS1	60	(S)	
NS1	65–70	IERILD/IERILK	IERILD
NS1	90–95	VTDMTP/MTDMTP/SP	VTDMTP/(VIDMTP)
NS1	111–114	FAGP	FAGP
NS1	124–127	IDKD/IDKE/(IDRE)	LDKN
NS1	152	S/N/G	S
NS1	162	(Q)	Q
NS1	194–197	(ISEA/ISEN)	
NS1	214–216	FAP/LTS/FTP/(LAP)	FAP
NS1	219–222	EQKL/KQKL/(EPKL)	ERKM
NEP	22–26	ESSSG/(VSSSG)	ESSSG
NEP	63–64	RN/KN/KS	
NEP	86–89	KLKT	KLKT

1 Geographical origin of the AIV segments is based on analyses by Wille et al. (2011) [5].

2 Signatures in brackets were only found in one or a few H13 or H16 sequences.

The polymerase (PB2, PB1 and PA) and M (M1 and M2) proteins were conserved across AIV subtypes and few signatures were found to be present (Table 1). Signatures highly specific for H13 and H16 viruses were identified in PB2 at positions 470, 559–560 and 674 (Table 1). In PB1 and PA, signatures were found in subgroups of Eurasian gull viruses (Table 1). In PB1-F2, only one signature proved to be specific for a few Eurasian H13 AIVs (Table 1). Similarly, the signatures in the M proteins were also mostly found in Eurasian gull viruses (Table 1). Specific amino acids that could be used to differentiate between Eurasian and American gull AIVs were found in PB2, NP and NS1 (Table 1).

The majority of H13 and H16 specific signatures were located in NS1, NP and NEP (Table 1). For most internal proteins, identical signatures were present in viruses of the H13 and the H16 subtype. In the initial analysis, variation in specific amino acids between H13 and H16 AIVs seemed to be present in NS1 at positions 52, 70, 90, 127, 197 and 215 (Figure S8) and in NEP at position 63 (Figure S9), respectively. However, validation against H13 and H16 protein sequences at NCBI showed that these were signatures characteristic for two subgroups of Eurasian H13 and H16 AIVs. One of these subgroups contained a large number of geographically reassorted American gull viruses.

### Prediction of protein similarity and localization

The *in silico* analysis predicted that a specific signature was present in NEP of the Eurasian H13 and H16 AIVs sequenced in this study. A motif with similarity to a conserved bacterial protein of unknown function (DUF2316), belonging to the Helix-Turn-Helix protein clan, was predicted in the Eurasian gull isolates by Pfam (independent E-value of 0.07), and was dependent on RN, KN or KS at positions 63–64 rather than GK, and on KLKT at positions 86–89. Changing these key positions of the H13 and H16 AIVs into Eurasian avian specific amino acids abolished the predicted function.

The PSORT analysis identified a nuclear localization signal (NLS) in the NS1 sequences of most AIVs (non H13 and H16 viruses), with a 7 amino acid pattern, starting at position 215 or 216. Neither H13 nor H16 NS1 sequences were predicted to harbor a NLS at this site. Further, in M1, the G residue at position 207 in H13 and H16 isolates abolished the predicted coiled-coil region at position 186–216 that were identified in most avian AIV M1 proteins. In M2, a signature at positions 55–58, located in the amphipathic helix within the C-terminal cytoplasmic domain [41], was observed in the Eurasian gull viruses.

## Discussion

In this paper, we searched for H13 and H16 specific signatures in the internal proteins that may be involved in host restriction of H13 and H16 viruses to gulls and shorebirds. The 15 AIVs used in the initial screening were isolated within a limited period of time (2005–2009) from scattered areas in Northern Eurasia. Eurasian gull AIVs were chosen for the initial analysis since a larger number of American gull viruses are reassortants [5]. The validity of the amino acids first identified as potentially H13 and H16 specific was subsequently assessed towards a large number of sequences in the NCBI database. Therefore, our approach should have been able to detect most signatures, including those present in American H13 and H16 viruses. The signatures were similar between H13 and H16 viruses, but might be biased towards the H13 subtype, since a lower number of complete H16 AIV genomes have been sequenced.

The final verification of the identified signatures against all available H13 and H16 sequences in the NCBI database was complicated by several factors. Firstly, a high proportion of reassorted gene segments were present among the H13 and H16 AIVs, causing a dilution effect on the H13 and H16 specific amino acids [5]. Moreover, several of the signatures were only found to be specific for subgroups of H13 and H16 AIVs. Secondly, adaptive mutations obtained through replication in a new or multiple host species or during virus cultivation, might be present in the studied viral sequences and have led to random changes in some of the signatures, and may explain why some of the H13 and H16 specific signatures were also exceptionally found in AIVs of other subtypes obtained from non-gull hosts. Thirdly, since the classical AIV sequences available from domestic poultry and waterfowls are more numerous and diverse than the H13 and H16 sequences from gulls, the importance of some H13 and H16 AIV signatures could be underestimated.

In the present study, signatures specific for H13 and H16 AIVs were identified in all nine internal viral proteins of the Eurasian gull AIVs. Since gulls are the primary hosts of AIVs of the H13 and H16 subtypes, some of the identified signatures could be important for host restriction, and may be responsible for the poor susceptibility of non-charadriiform avian species to these virus subtypes [42], [43]. Among internal proteins, PB2, NP, NS1 and NEP contained most signatures. This is consistent with findings by Fouchier et al. (2005) who reported that European gull viruses can be phylogenetically distinguished from other influenza A viruses based on their PB2, NP and NS genes [3].

We found H13 and H16 specific signatures at positions 470, 559, 560 and 674 in PB2. These sites have previously been reported to contain amino acids unique for H13 gull PB2s [21]. Our analysis found amino acids VI to be common at positions 559–560 in addition to MI [21], and that MI and VI predominated in American and Eurasian gull AIVs, respectively.

Few H13 and H16 specific signatures were present in PB1 and PA, which is in accordance with previous findings [44]. All sequenced PA and PB1 segments in American AIVs, as of today, have been of Eurasian origin [45] explaining why we only found H13 and H16 specific signatures in a selection of the Eurasian viruses. In Europe, most sequenced H13 and H16 genomes are composed exclusively of gull-like segments. In America, where shorebirds have been suggested to play an important role in the AIV dynamics [4], H13 and H16 AIVs contain avian-like polymerase segments [5]. A polymerase with avian-like PB1 and PA subunits may increase the overall viral fitness of the American gull viruses and facilitate virus transmission between gulls and shorebirds. Gulls and shorebirds belong to the same avian order (i.e. Charadriiformes), which may ease the transmission of H13 and H16 AIVs between these birds. Also in the other viral proteins, a lower number of H13 and H16 specific signatures were found in the American viruses compared to that in the Eurasian viruses. This may be explained by transmission in different host species within each continent, or alternatively represent viral adaptations to genetically different bird populations.

In this study, the M proteins were shown to be conserved across AIV subtypes as previously reported [46], suggesting a minor role of the M proteins in host restriction of AIVs between avian species. In contrast, previous studies have indicated that several amino acid residues in NP are important for the host specificity of H13 AIVs [23]. We identified six signatures in NP of which several had already been described as H13-specific [23]. In addition to the H13-specific 350T, 353L, 375N and 433P reported by Gorman et al. (1990) [23], we found that 350–353 ARVL/TRVL, 375 S/N and 432–433 SA/NP were specific for Eurasian and American H13 and H16 viruses, respectively. NP participates in NP-NP interactions, plays a role during transcription and binds to RNA, PB1, PB2, M1 and to several cellular proteins that are important for nuclear import and export of ribonucleoprotein [47], [48]. It was recently shown that NP of human influenza A viruses determines the sensitivity of Mx proteins [49]. The antiviral activity of Mx proteins varies between and within species, and a wide range of intra- and interspecies variation in the Mx gene has been found in wild ducks [50]. Different signatures in NP may contribute to the host restriction of H13 and H16 AIVs to gulls.

Our results show that most H13 and H16 AIV specific signatures are present in the NS1 protein [51]. Some of these signatures are located in the N-terminal RNA binding domain whereas the majority are found in the C-terminal effector domain. The NS1 protein is only present in infected cells [51] and its major function is to counter the innate host antiviral defense primarily by antagonizing type I interferon (IFN) response. Both the RNA binding domain and the effector domain of NS1 are required for optimal suppression of the host immune responses during influenza infection [52]. NS1 proteins of influenza A viruses have multiple ways to exert the IFN-antagonism and differences in these actions have been found to be strain-specific [53]. This suggests that the H13 and H16 AIVs have developed specific signatures in the NS1 protein that enable them to replicate in gulls. Furthermore, the NS1 proteins of H13 and H16 AIVs may be less adapted to suppress the innate immune response in ducks and thus not be able to efficiently replicate in duck cells.

The effector domain of the NS1 protein facilitates viral replication by interaction with proteins involved in innate cellular antiviral response [54]. One of the H13 and H16 AIV specific signatures was located in the effector domain at amino acid positions 214–216. In a previous study a Src homology (SH) binding motif, PPLPPK, called SH3, was mapped at NS1 amino acid positions 212–217 of most AIVs subtypes [54], [55]. This motif is essential for binding to cellular CRK and CRKL proteins, and P212, P215 and K217 are critical for this binding [55]. Binding ability of NS1 to CRK/CRKL may have evolved in virus strains that over-activate the cellular JNK–ATF2 signaling pathway leading to apoptosis [54]. Thus, AIVs containing PPLPPK replicate more efficiently. Our database searches confirm that the SH3 motif, PPLPPK, is conserved among the NS1 proteins of most AIV subtypes. However, this SH3 motif was not found in H13 and H16 AIVs, and it is also uncommon in human influenza A viruses [54]. In human influenza viruses the P in position 215 of NS1 is replaced by T, which results in failure to bind CRK/CRKL proteins [55]. In the NS1 of H13 and H16 AIVs, we found that T or A were present at position 215. T215 in human influenza A viruses is phosphorylated, and replacement with an A residue reduces viral replication [56]. The disrupted SH3 motifs in H13 and H16 AIVs suggest that their NS1s also lack CRK/CRKL binding activity.

NS1 has previously been shown to harbor one conserved NLS, NLS1, at position 34–38. In addition, several influenza viruses harbor a second NLS, NLS2, in the C-terminal part of the NS1 protein (216–221 PKQKRK). Both NLSs are involved in binding α-importin, which facilitates nuclear import [57]–[59]. We found the predicted NLS2 in most avian NS1 proteins, involving parts of the 214–216 and the 219–222 signatures, while NLS2 was not present in the NS1 protein of Eurasian H13 and H16 AIVs. However, as in other AIVs, NLS1 is conserved in the H13 and H16 viruses, suggesting that NLS1 is sufficient for nuclear import of NS1 in these viruses.

For most internal viral proteins, the signatures were found to be similar among H13 and H16 viruses. However, different signatures between the H13 and the H16 subtype seemed to be present in NS1 and NEP in the initial analysis. It has previously been shown that all the internal genes of H13 and H16 viruses from European black-headed gull (*Chroicocephalus ridibundus*), except NS, are closely related [3]. Our initial analysis only included two complete H16 genomes. In addition, one of the selected H13N8 viruses in the initial *in silico* screening, isolated from herring gull (*Larus argentatus*) in Mongolia, had the same motifs as the H16 viruses. However, these features are shared by most NS1 proteins from H16 sequences deposited in GenBank, while only few of the H13 sequences have these combinations of amino acids in NS1. The signature patterns in NS1 turned out to be characteristic for two subgroups of Eurasian H13 and H16 AIVs, of which one contained a large number of American reassorted gull viruses. These results suggest that different genotypes of NS1 are present in these two groups of H13 and H16 AIVs. Alternatively, it might reflect reassortment between H13 and H16 AIVs.

The NS1 is a multifunctional viral protein implying that a multifaceted selection pressure is exerted on this protein, which is mirrored in a large nucleotide sequence variation [51]. The multifunctionality might also bring along strain variations and host-specific adaptation.

In this study, we examined the internal proteins of H13 and H16 AIVs for presence of specific signatures. The surface proteins, the HA in particular, are important determinants of host specificity of influenza A viruses [20]. The HAs of H13 and H16 AIVs have been found to be resistant to cleavage by several proteases including trypsin, impairing further fusion process *in vitro* [60], [61]. The crystal structure of H16 HA0 shows the presence of an α-helix covering the cleavage site which may be involved in the protease resistance [61]. Furthermore, it has previously been reported that H13 and H16 AIVs replicate poorly in Madin Darby Canine Kidney (MDCK) cells in the presence of trypsin, but efficiently in embryonated chicken eggs, the latter also as recombinants with H13 or H16 subtypes in an A/PR/8/34 backbone, when cell suspension rather than supernatant is inoculated [62]. The presence of HA activating proteases in target species is thus likely an important factor in the host range of influenza A viruses [60]. The identified signatures in our study indicate that the host specificity of H13 and H16 AIVs may not solely be determined by receptor recognition and interaction between HA and host proteases, but that it may be partially explained by the internal viral proteins, and NS1 and NP in particular.

## Conclusions

To summarize, several H13 and H16 AIV specific amino acid signatures were identified in the internal proteins by bioinformatics analysis. The NS1 and NP proteins contained most specific signatures. In particular, H13 and H16 AIVs were predicted to lack a NLS in NS1 present in AIVs of other subtypes. The number of available H13 and H16 AIV sequences in the public databases was much lower compared to that from other AIV subtypes. Sequence data from more geographically dispersed areas is therefore needed to generalize the importance of the identified signatures. Further experimental data will be needed to confirm the *in silico* proposed signatures, and our results provide a basis for future functional studies to get a better understanding of host adaptation and epidemiology of AIVs in wild birds.

## Supporting Information

Figure S1 **Amino acid heterogeneity in the PB2 protein.** Amino acid heterogeneity in the PB2 protein of the 15 Eurasian avian (non H13 and H16) and Eurasian gull (H13 and H16) avian influenza viruses (AIVs) included in the initial analysis. Reassorted gene segments of Eurasian avian origin are indicated by two asterisks.
(TIF)

Figure S2 **Amino acid heterogeneity in the PB1 protein.** Amino acid heterogeneity in the PB1 protein of the 15 Eurasian avian (non H13 and H16) and Eurasian gull (H13 and H16) avian influenza viruses (AIVs) included in the initial analysis.
(TIF)

Figure S3 **Amino acid heterogeneity in the PB1-F2 protein.** Amino acid heterogeneity in the PB1-F2 protein of the 15 Eurasian avian (non H13 and H16) and Eurasian gull (H13 and H16) avian influenza viruses (AIVs), included in the initial analysis.
(TIF)

Figure S4 **Amino acid heterogeneity in the PA protein.** Amino acid heterogeneity in the PA protein of the 15 Eurasian avian (non H13 and H16) and Eurasian gull (H13 and H16) avian influenza viruses (AIVs) included in the initial analysis. Reassorted gene segments of Eurasian avian origin are indicated by two asterisks.
(TIF)

Figure S5 **Amino acid heterogeneity in the NP protein.** Amino acid heterogeneity in the NP protein of the 15 Eurasian avian (non H13 and H16) and Eurasian gull (H13 and H16) avian influenza viruses (AIVs) included in the initial analysis.
(TIF)

Figure S6 **Amino acid heterogeneity in the M1 protein.** Amino acid heterogeneity in the M1 protein of the 15 Eurasian avian (non H13 and H16) and Eurasian gull (H13 and H16) avian influenza viruses (AIVs) included in the initial analysis.
(TIF)

Figure S7 **Amino acid heterogeneity in the M2 protein.** Amino acid heterogeneity in the M2 protein of the 15 Eurasian avian (non H13 and H16) and Eurasian gull (H13 and H16) avian influenza viruses (AIVs) included in the initial analysis.
(TIF)

Figure S8 **Amino acid heterogeneity in the NS1 protein.** Amino acid heterogeneity in the NS1 protein of the 15 Eurasian avian (non H13 and H16) and Eurasian gull (H13 and H16) avian influenza viruses (AIVs) included in the initial analysis.
(TIF)

Figure S9 **Amino acid heterogeneity in the NEP protein.** Amino acid heterogeneity in the NEP protein of the 15 Eurasian avian (non H13 and H16) and Eurasian gull (H13 and H16) avian influenza viruses (AIVs) included in the initial analysis.
(TIF)

Table S1 Primers used in the PCR amplification of PB1 from AIV H13N2* and H9N2** and of PB2 from AIV H3N8***.
(DOCX)

Table S2 **Primers used for internal sequencing of the PA, PB1 and PB2 genes.**
(DOCX)

Table S3 Accession numbers for complete AIV genomes from gulls in Eurasia, available from the Influenza Virus Resource at NCBI as of February 2012*.
(DOC)
