# Supplementary figures and images for: Host Restrictions of Avian Influenza Viruses: In Silico Analysis of H13 and H16 Specific Signatures in the Internal Proteins
Source: PLoS One. 2013 Apr 30;8(4):e63270. doi: 10.1371/journal.pone.0063270 (PMC3639990; doi:10.1371/journal.pone.0063270)

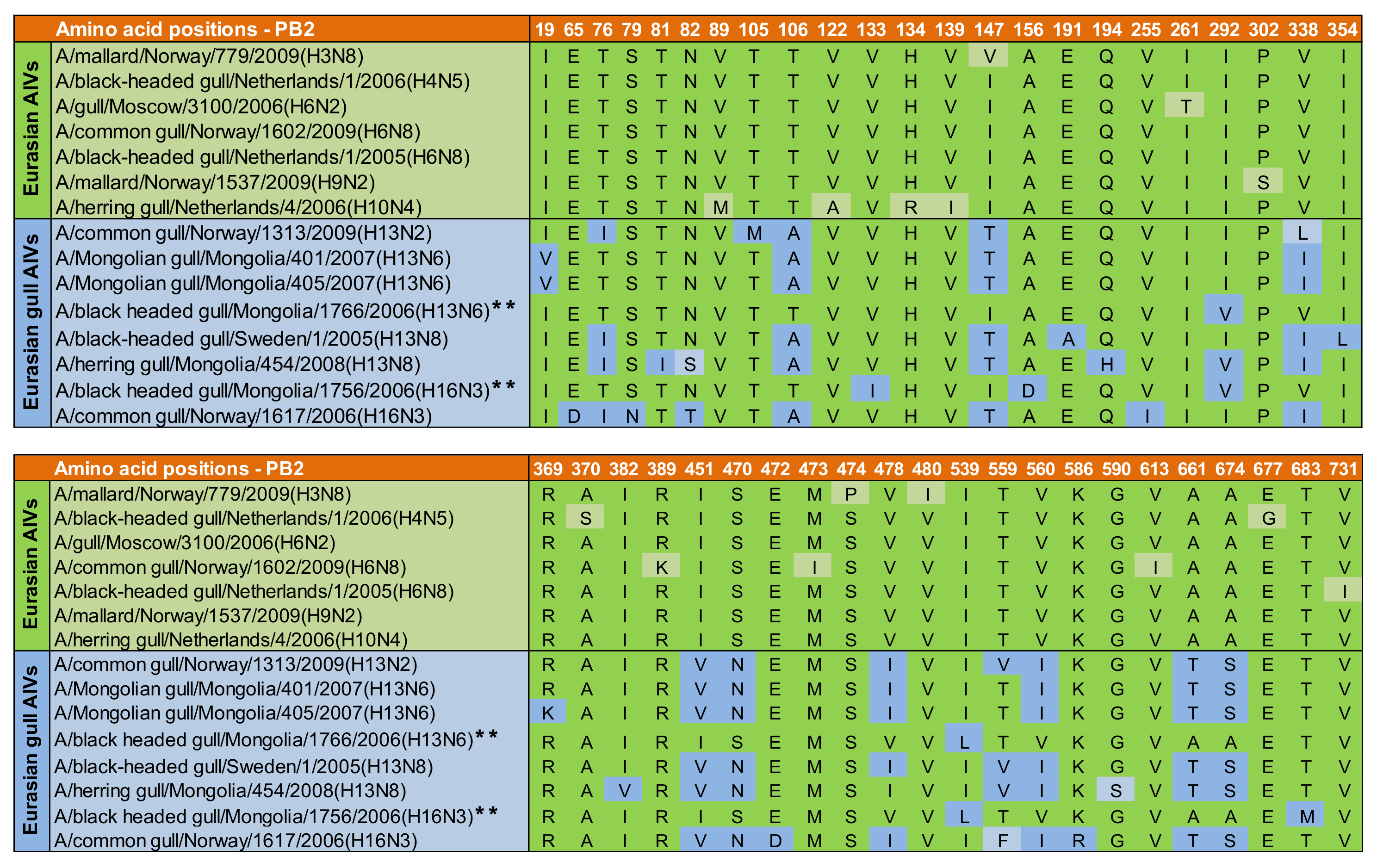

Supplement: Figure S1 — Amino acid heterogeneity in the PB2 protein. Amino acid heterogeneity in the PB2 protein of the 15 Eurasian avian (non H13 and H16) and Eurasian gull (H13 and H16) avian influenza viruses (AIVs) included in the initial analysis. Reassorted gene segments of Eurasian avian origin are indicated by two asterisks. (TIF) [file pone.0063270.s001.tif]

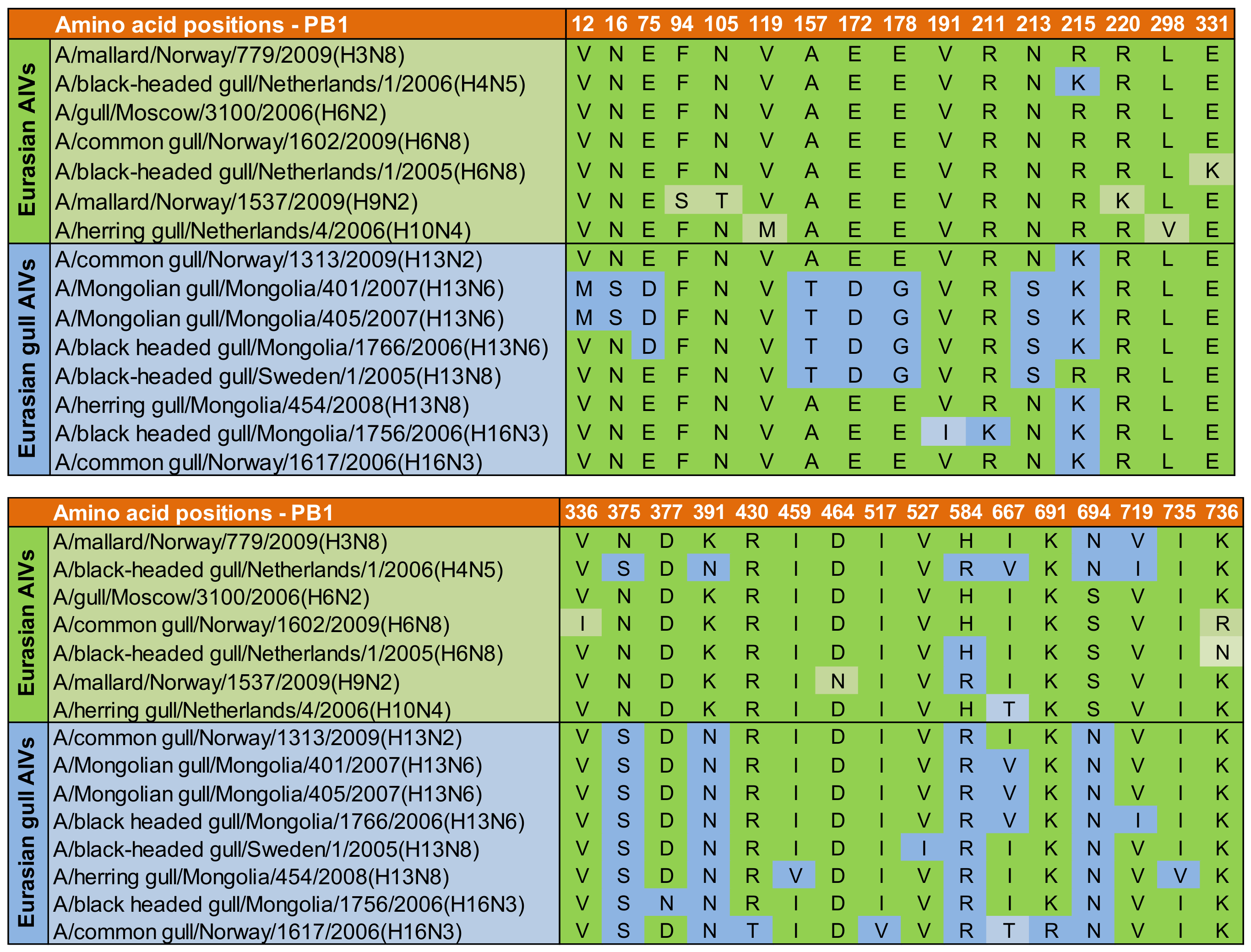

Supplement: Figure S2 — Amino acid heterogeneity in the PB1 protein. Amino acid heterogeneity in the PB1 protein of the 15 Eurasian avian (non H13 and H16) and Eurasian gull (H13 and H16) avian influenza viruses (AIVs) included in the initial analysis. (TIF) [file pone.0063270.s002.tif]

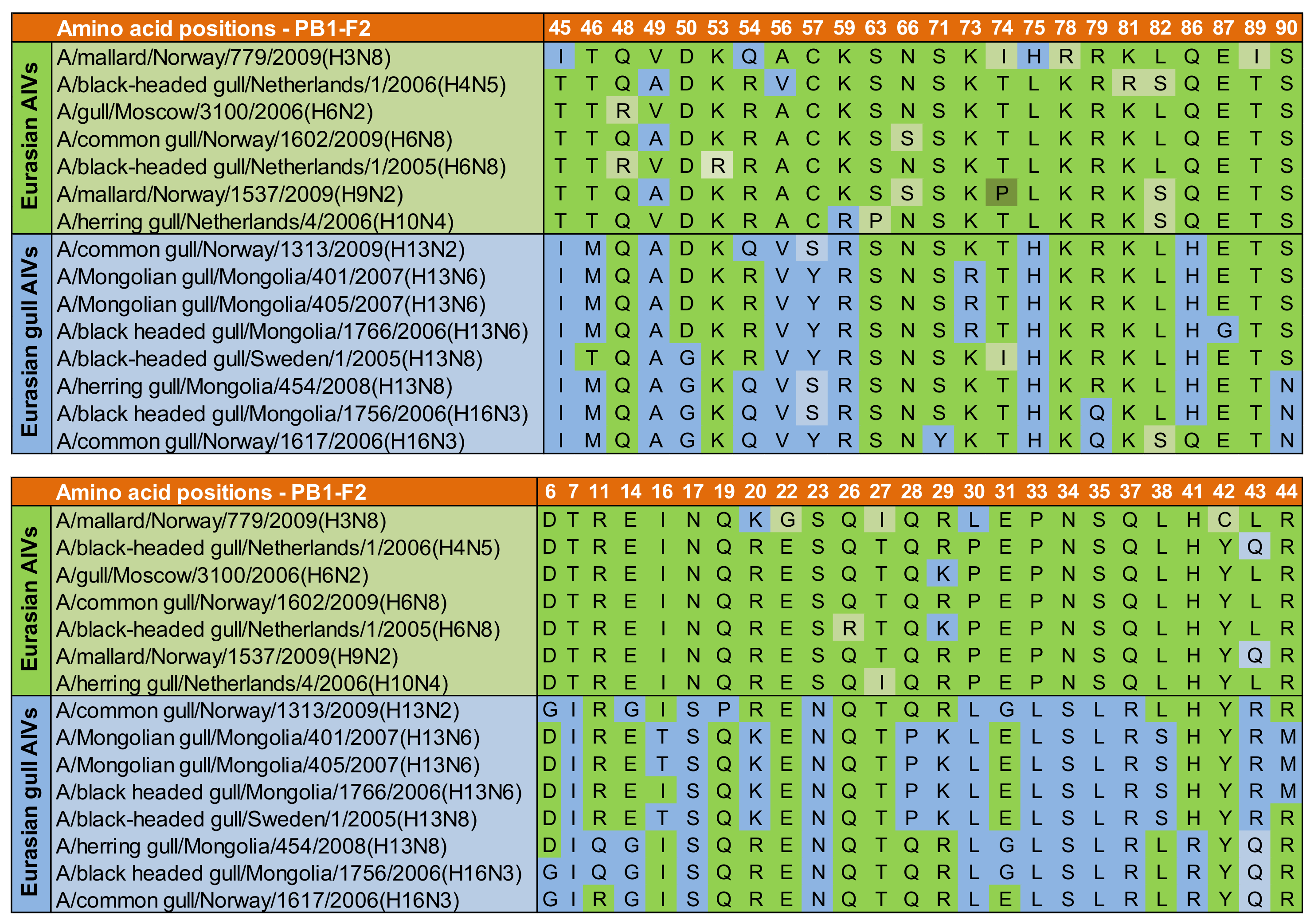

Supplement: Figure S3 — Amino acid heterogeneity in the PB1-F2 protein. Amino acid heterogeneity in the PB1-F2 protein of the 15 Eurasian avian (non H13 and H16) and Eurasian gull (H13 and H16) avian influenza viruses (AIVs), included in the initial analysis. (TIF) [file pone.0063270.s003.tif]

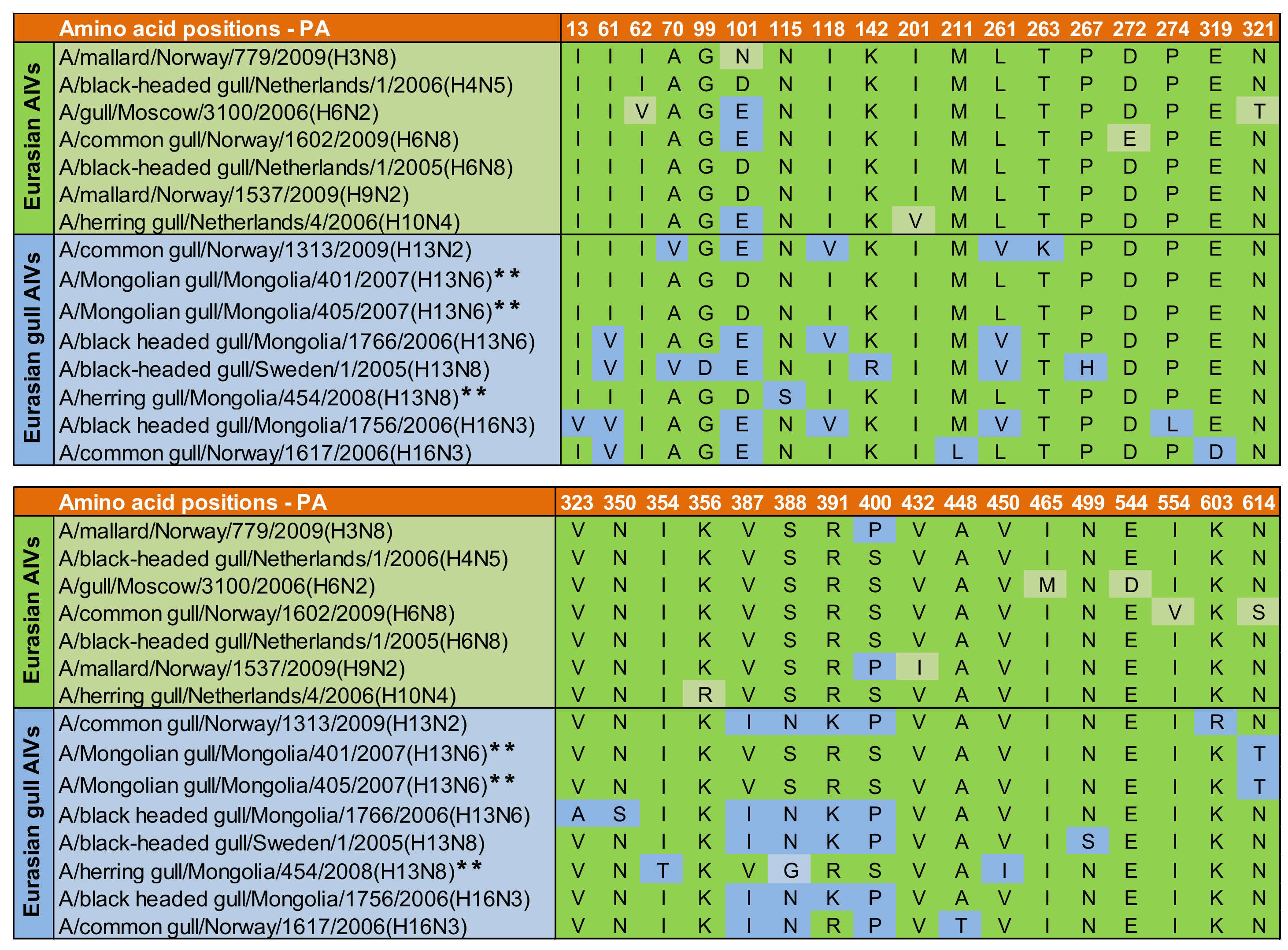

Supplement: Figure S4 — Amino acid heterogeneity in the PA protein. Amino acid heterogeneity in the PA protein of the 15 Eurasian avian (non H13 and H16) and Eurasian gull (H13 and H16) avian influenza viruses (AIVs) included in the initial analysis. Reassorted gene segments of Eurasian avian origin are indicated by two asterisks. (TIF) [file pone.0063270.s004.tif]

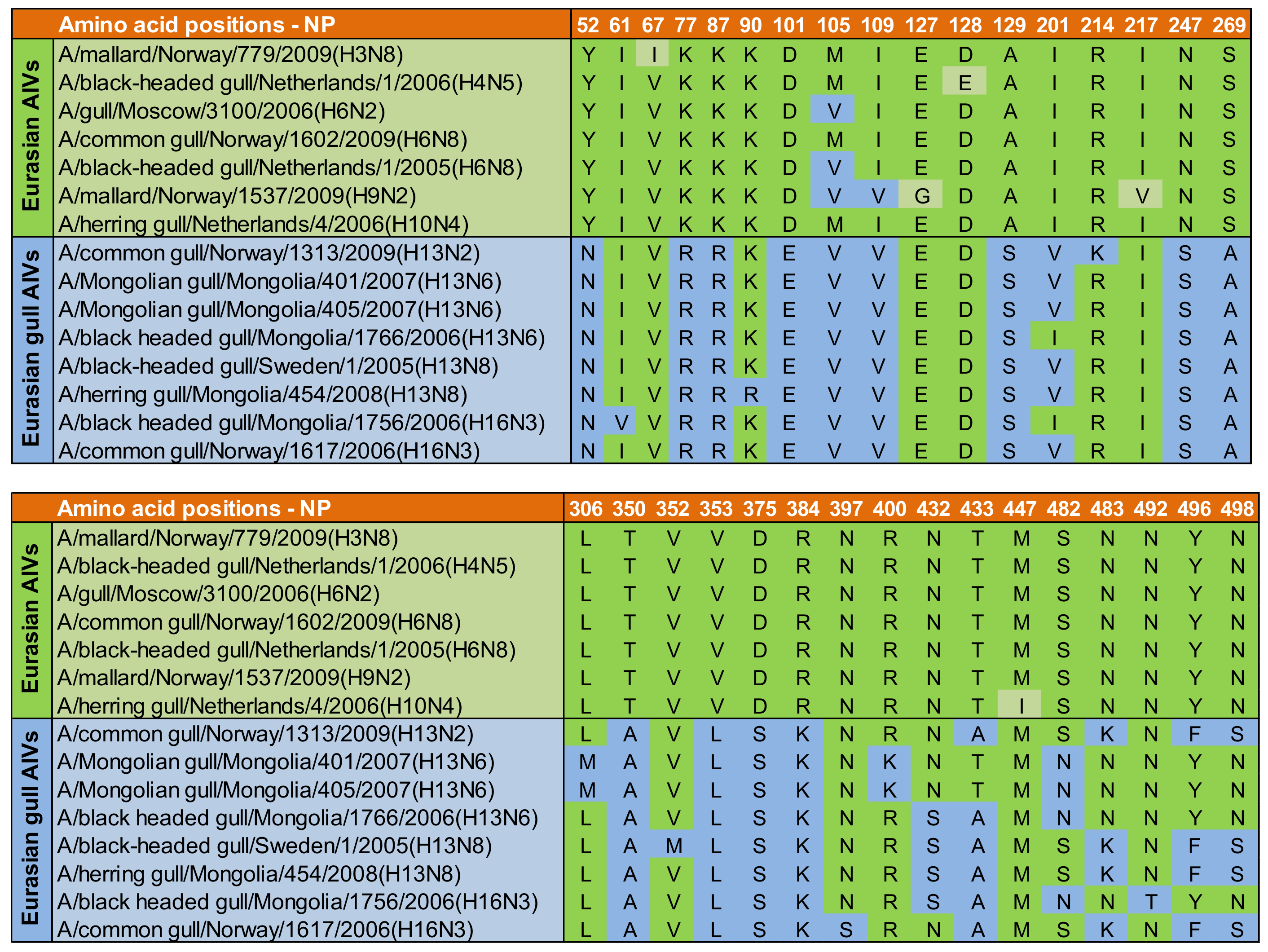

Supplement: Figure S5 — Amino acid heterogeneity in the NP protein. Amino acid heterogeneity in the NP protein of the 15 Eurasian avian (non H13 and H16) and Eurasian gull (H13 and H16) avian influenza viruses (AIVs) included in the initial analysis. (TIF) [file pone.0063270.s005.tif]

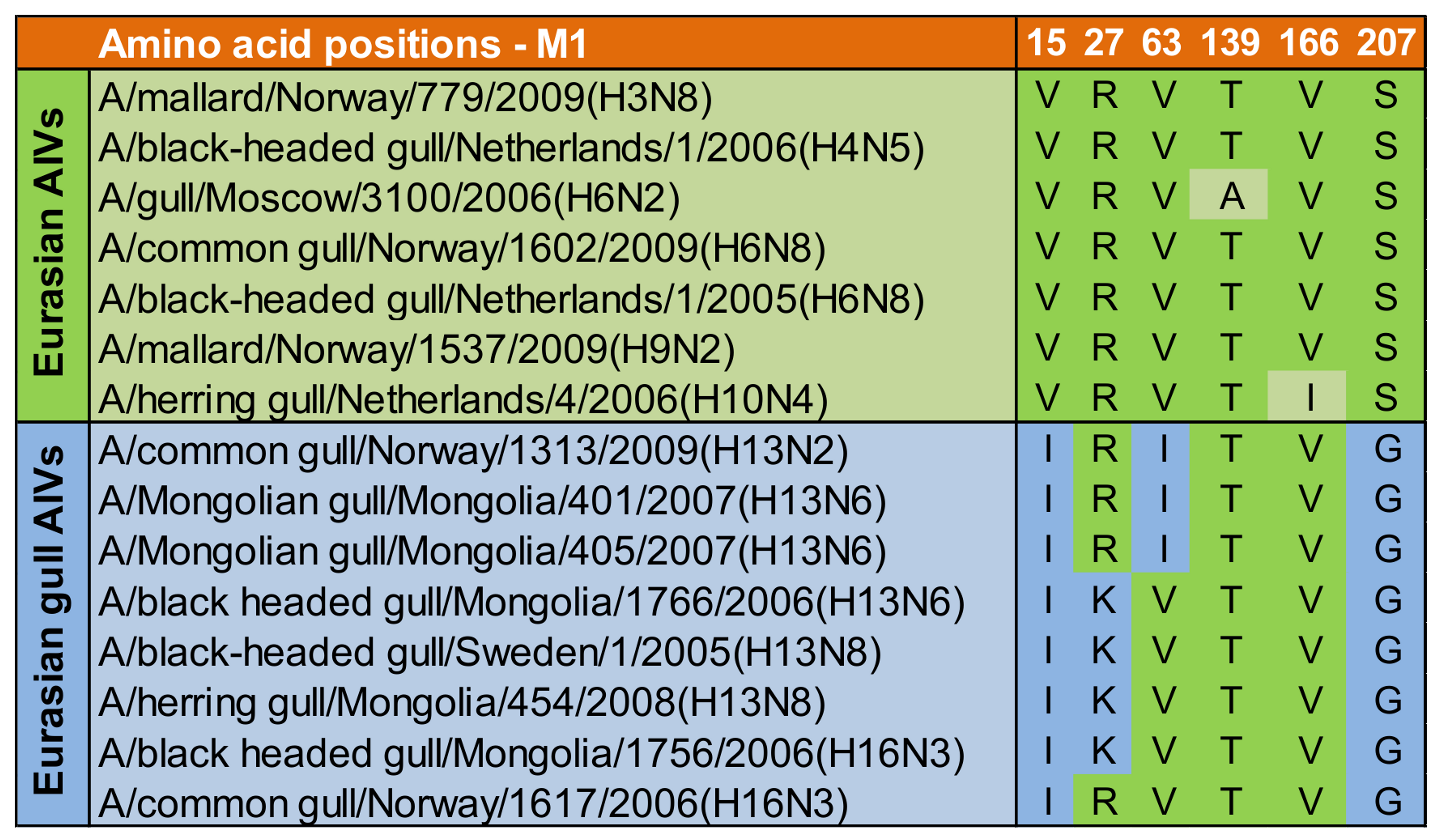

Supplement: Figure S6 — Amino acid heterogeneity in the M1 protein. Amino acid heterogeneity in the M1 protein of the 15 Eurasian avian (non H13 and H16) and Eurasian gull (H13 and H16) avian influenza viruses (AIVs) included in the initial analysis. (TIF) [file pone.0063270.s006.tif]

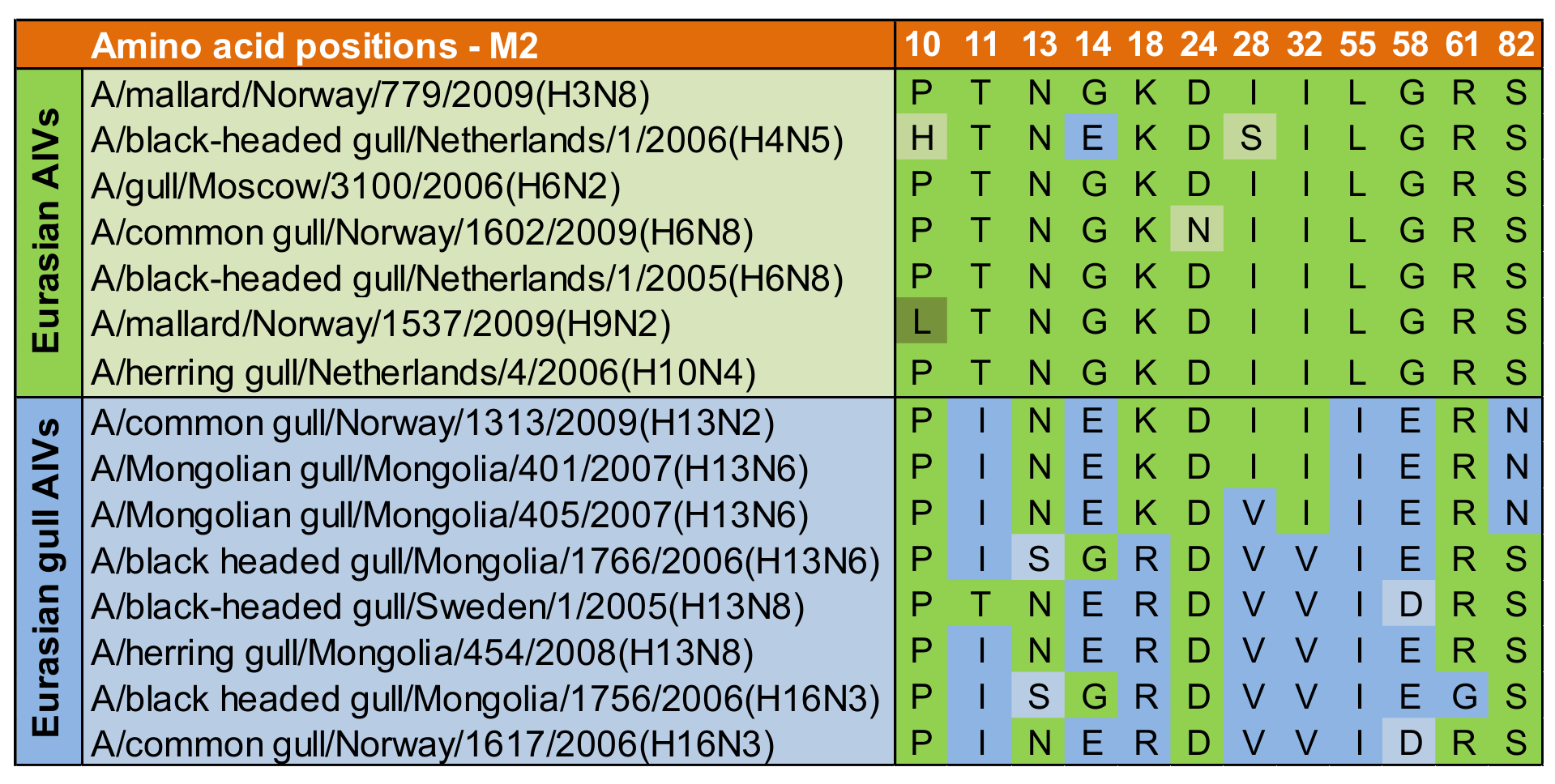

Supplement: Figure S7 — Amino acid heterogeneity in the M2 protein. Amino acid heterogeneity in the M2 protein of the 15 Eurasian avian (non H13 and H16) and Eurasian gull (H13 and H16) avian influenza viruses (AIVs) included in the initial analysis. (TIF) [file pone.0063270.s007.tif]

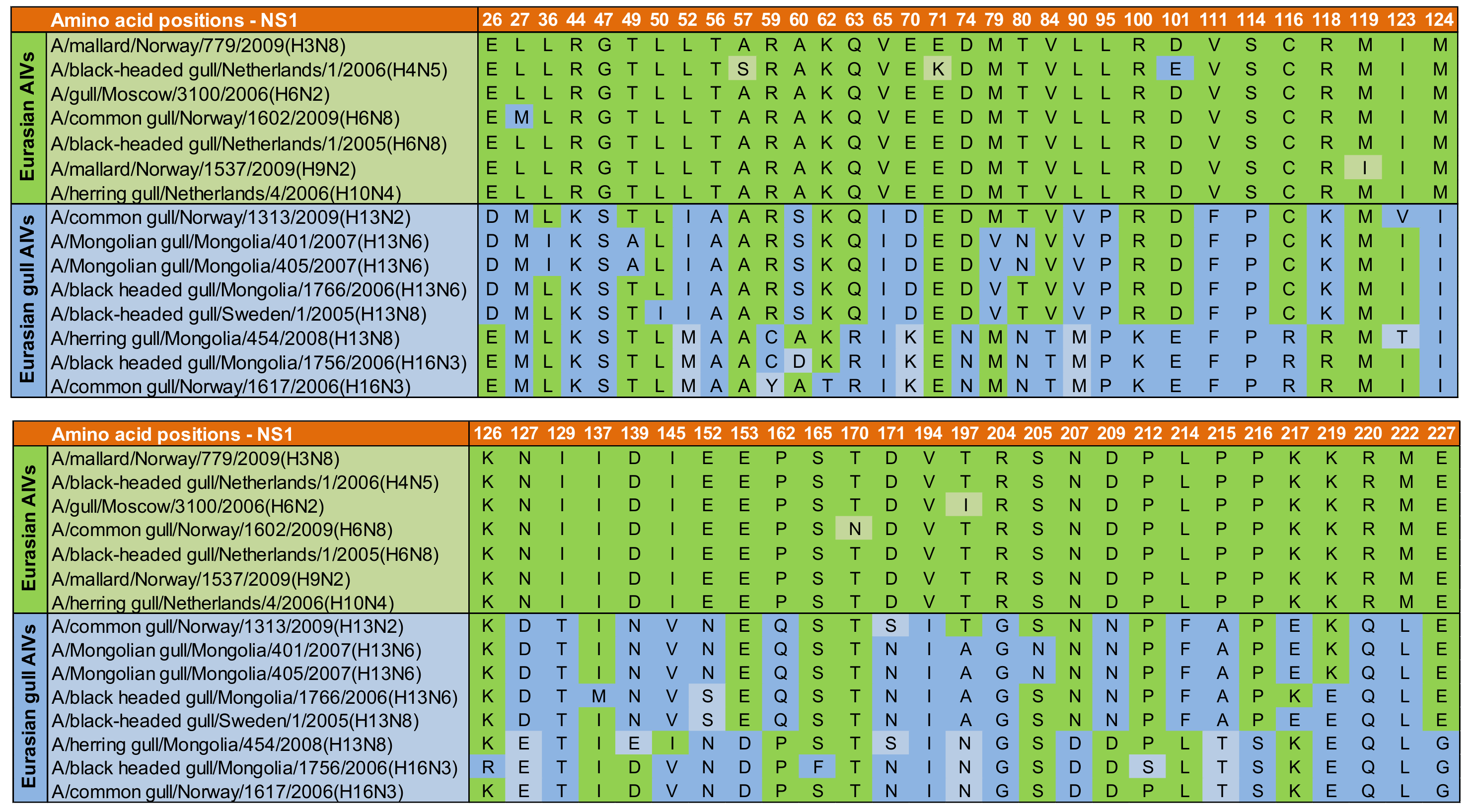

Supplement: Figure S8 — Amino acid heterogeneity in the NS1 protein. Amino acid heterogeneity in the NS1 protein of the 15 Eurasian avian (non H13 and H16) and Eurasian gull (H13 and H16) avian influenza viruses (AIVs) included in the initial analysis. (TIF) [file pone.0063270.s008.tif]

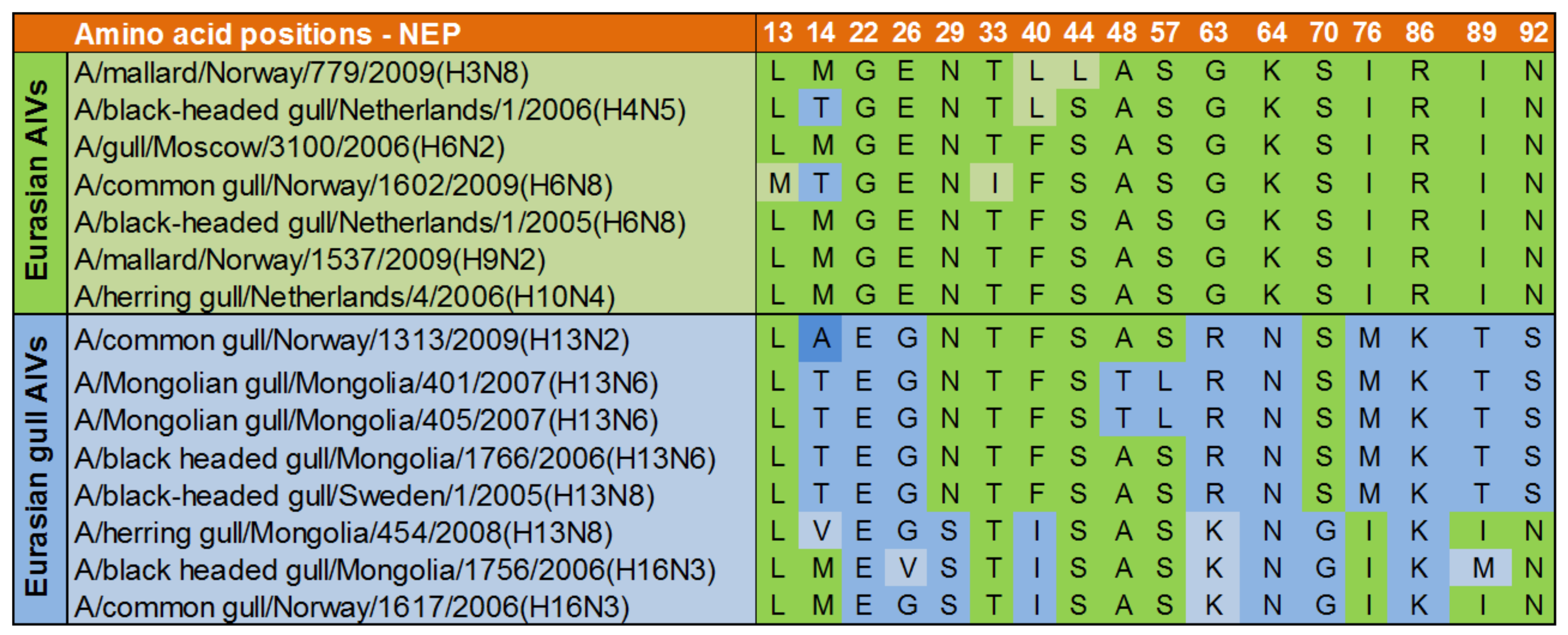

Supplement: Figure S9 — Amino acid heterogeneity in the NEP protein. Amino acid heterogeneity in the NEP protein of the 15 Eurasian avian (non H13 and H16) and Eurasian gull (H13 and H16) avian influenza viruses (AIVs) included in the initial analysis. (TIF) [file pone.0063270.s009.tif]
