# Supplementary material for: Host Restrictions of Avian Influenza Viruses: In Silico Analysis of H13 and H16 Specific Signatures in the Internal Proteins
Source: PLoS One. 2013 Apr 30;8(4):e63270. doi: 10.1371/journal.pone.0063270 (PMC3639990; doi:10.1371/journal.pone.0063270)
Supplement: Table S1 — Primers used in the PCR amplification of PB1 from AIV H13N2* and H9N2** and of PB2 from AIV H3N8***. (DOCX) [file pone.0063270.s010.docx]

**Table S1.** Primers used in the PCR amplification of PB1 from AIV H13N2* and H9N2** and of PB2 from AIV H3N8***.

| **Segment** | **Forward primer (5’- 3’)** | **Reverse primer (5’- 3’)** |
| --- | --- | --- |
| 1 (PB2) | PB2_fwd_1: GGGAGCGAAAGCAGGTCAA | PB2_rev_1632: TCAGGACCATTGATCTCCCACA |
|  | PB2_fwd_1414: AATGTAATGGGAATGATCGGAATA | PB2_rev_2309: CTCGTATTAGTAGAAACAAGGTCGTTT |
| 2 (PB1) | PB1_fwd_1: AGCGAAAGCAGGCAAACCATT | PB1_rev_691: GCATCTTTGGTCATTGTGTTCAGTGT |
|  | PB1_fwd_617: AAATGGTCACACAAAGAACAATAGG | PB1_rev_1395: TCCACTCCTGCTTGTATTCCC |
|  | PB1_fwd_1253: TGAGTCCTGGAATGATGATGGG | PB1_rev_2309: AAATGCCTTGTTCTACTAATACGAGACG |

*A/common gull/Norway/1313/2009(H13N2)

**A/mallard/Norway/1537/2009(H9N2)

*** A/mallard/Norway/779/2009(H3N8)
