# Supplementary material for: Host Restrictions of Avian Influenza Viruses: In Silico Analysis of H13 and H16 Specific Signatures in the Internal Proteins
Source: PLoS One. 2013 Apr 30;8(4):e63270. doi: 10.1371/journal.pone.0063270 (PMC3639990; doi:10.1371/journal.pone.0063270)
Supplement: Table S2 — Primers used for internal sequencing of the PA, PB1 and PB2 genes. (DOCX) [file pone.0063270.s011.docx]

**Table S2.** Primers used for internal sequencing of the PA, PB1 and PB2 genes.

| **Segment** | **Forward primers (5’- 3’)** | **Reverse primers (5’- 3’)** |
| --- | --- | --- |
| 1 (PB2) | CAGGAGGGGAGGTGAGAAATGATG | GTTCTCTGTTCCCTGTGTTTC |
| 2 (PB1) | CCTGCCTTTGAATGGGTCTGCT | AGAGGCAAATTGAAGAGGCGGGC |
| 3 (PA) | TGAAAAAAACACCACGCCCTCT | GCAATGTGTTCAATAGGGGCAAC |
