# Supplementary material for: Host Restrictions of Avian Influenza Viruses: In Silico Analysis of H13 and H16 Specific Signatures in the Internal Proteins
Source: PLoS One. 2013 Apr 30;8(4):e63270. doi: 10.1371/journal.pone.0063270 (PMC3639990; doi:10.1371/journal.pone.0063270)
Supplement: Table S3 — Accession numbers for complete AIV genomes from gulls in Eurasia, available from the Influenza Virus Resource at NCBI as of February 2012*. (DOC) [file pone.0063270.s012.doc]

**Table S3**. Accession numbers for complete AIV genomes from gulls in Eurasia, available from the Influenza Virus Resource at NCBI as of February 2012^*^.

| **Accession numbers** (Segment 1-8) | **Virus isolates** |
| --- | --- |
|
| CY076992-99 | A/black-headed gull/Netherlands/1/2006(H4N5) |
| EU152234-41 | A/gull/Moscow/3100/2006(H6N2) |
| CY041378-85 | A/black-headed gull/Netherlands/1/2005(H6N8) |
| CY077032-39 | A/herring gull/Netherlands/4/2006(H10N4) |
| GQ907310-17 | A/Mongolian gull/Mongolia/401/2007(H13N6) |
| GQ907318-25 | A/Mongolian gull/Mongolia/405/2007(H13N6) |
| GQ907302-9 | A/black headed gull/Mongolia/1766/2006(H13N6) |
| CY077000-7 | A/black-headed gull/Sweden/1/2005(H13N8) |
| JF775470-77 | A/herring gull/Mongolia/454/2008(H13N8) |
| GQ907294-301 | A/black headed gull/Mongolia/1756/2006(H16N3) |

^*^ Three genomes were excluded from the analysis (not shown).
